# p53 Affects PGC1α Stability Through AKT/GSK-3β to Enhance Cisplatin Sensitivity in Non-Small Cell Lung Cancer

**DOI:** 10.3389/fonc.2020.01252

**Published:** 2020-08-21

**Authors:** Xinyue Deng, Yang Li, Shuang Gu, Yingying Chen, Bingbing Yu, Jing Su, Liankun Sun, Yanan Liu

**Affiliations:** ^1^Key Laboratory of Pathobiology, Ministry of Education, Department of Pathophysiology, College of Basic Medical Sciences, Jilin University, Changchun, China; ^2^Department of Thoracic Surgery, Jilin Provincial People's Hospital, Changchun, China

**Keywords:** non-small cell lung cancer, p53, PGC1α, mitochondrial function, AKT, GSK-3β, CDDP

## Abstract

Drug resistance greatly limits the therapeutic efficacy of treatment of non-small cell lung cancer (NSCLC). One of the important factors is the dysfunction of tumor suppressor p53. Recent studies have suggested that p53 suppresses tumors by regulating number of mitochondrial proteins, including peroxisome proliferator-activated receptor coactivator (PGC1α). Although several studies have confirmed the interaction between p53 and PGC1α, the precise mechanism has not been completely determined in NSCLC. In this study, we investigated the specific signaling between p53 and PGC1α to improve anti-tumor drug effects on NSCLC. We found that low expression of p53 and high expression of PGC1α correlated with shorter survival time of NSCLC patients. *In vitro* experiments confirmed that NCI-H1299 (p53-null) cells had high levels of PGC1α and were insensitive to cisplatin (CDDP). When PGC1α was knocked down, the sensitivity to cisplatin was increased. Notably, the stability of PGC1α is an important mechanism in its activity regulation. We demonstrated that p53 decreased the stability of PGC1α via the ubiquitin proteasome pathway, which was mediated by protein kinase B (AKT) inhibition and glycogen synthase kinase (GSK-3β) activation. Therefore, p53 may regulate the stability of PGC1α through the AKT/GSK-3β pathway, thus affect the chemosensitivity of NSCLC.

## Background

Lung cancer is one of the most malignant tumors in the world, and the main type is non-small cell lung cancer (NSCLC). In recent years, drug resistance has become a problem in the treatment of NSCLC, leading to poor prognosis (1). Tumor suppressor p53, a major defense factor against cancer, initiates apoptosis by triggering a caspase cascade (2). However, about half of the NSCLC subtypes have p53 missense mutations, resulting in loss of wild type p53 activity (3, 4). Tumors with loss of p53 function are often resistant to chemotherapy drugs (5–7). Therefore, for NSCLC patients with p53 dysfunction, seeking new targeted treatment has become the key to overcoming chemoresistance.

p53 regulates many cellular functions, including cell cycle arrest, senescence and apoptosis, to inhibit tumorigenesis (8). Recent studies have found that it is also involved in the regulation of tumor suppression though other functions, such as metabolic reprogramming, and antioxidant, and mitochondrial function regulation. An increasing number of mitochondrial proteins involved in mitochondrial metabolism and respiration are regulated by p53 (9, 10). Peroxisome proliferator-activated receptor coactivator (PGC1α), a master regulator of mitochondrial function, mediates mitochondrial biogenesis, oxidative phosphorylation, and mitochondrial reactive oxygen species (ROS) detoxification. Several studies have confirmed the interaction between p53 and PGC1α (11), however, its regulatory mechanism has not been completely determined. Aquilano et al. have found that p53 binds to the promoter region of *PPARGC1A* to induce its expression, and depletion of the antioxidant factor, glutathione, induces the p53-PGC1α-Nuclear factor 2 (NRF2) axis (12). However, Villeneuve et al. have demonstrated that p53 inhibits PGC1α and induces oxidative stress in cardiomyocytes (13). Additionally, PGC1α plays an important role in tumor chemotherapy drug resistance. Upregulation of PGC1α protected tumor cells from cisplatin (CDDP) cytotoxicity by regulating mitochondrial respiratory chain complex proteins and the oxygen consumption rate (OCR) in colon cancer (14). Gao et al. have also found that targeting PGC1α reduced the drug resistance of melanoma to mitogen-activated protein kinase (MAPK) inhibitors (15). Taken together, we speculated that the regulatory relationship between p53 and PGC1α is an important drug resistance mechanism of NSCLC.

Owing to the short half-life of PGC1α, its stability regulation is an important mechanism of its activity regulation (16). PGC1α activity is modulated by both expression and posttranscription modifications. Rozalyn et al. have found that PGC1α degradation by the proteasome system depends on glycogen synthase kinase (GSK-3β)-mediated phosphorylation (17). Additionally, the serine/threonine-specific kinase, Akt, plays an important role in a variety of cellular processes. After activation, Akt is transferred to different subcellular compartments to phosphorylate the multifunctional serine/threonine-specific kinase, GSK-3β, to inhibit its activity (18). Previous studies have found that p53 inhibits the proliferation and metastasis of osteosarcoma by inhibiting the PI3K/AKT/mTOR pathway (19). Rueda-Rincon et al. have also confirmed that p53 affects cell survival by inhibiting the oncogenic AKT pathway (20). Thus, we speculated that p53 affects the stability of PGC1α through the AKT/GSK-3β pathway.

Here, we investigated whether p53 regulates the stability of PGC1α through the AKT/GSK-3β pathway, and thus affects the chemosensitivity of tumor cells. Our results showed that low p53 expression and high PGC1α expression correlated with poor survival rate. Furthermore, p53 affected mitochondrial biosynthesis by regulating PGC1α to reduce chemoresistance of NSCLC. Moreover, our results indicated that PGC1α may be a potential target for individualized treatment of patients with different p53 backgrounds.

## Methods and Materials

### Reagents and Antibodies

The human non-small lung cancer cell lines, A549, H1975, and H1299, were obtained from the cell bank of the Institute of Biochemistry and Cell Biology (Shanghai, China). A549 cells were cultured in F-12K medium, and H1975 and H1299 cells were cultured in RPMI-1640 medium (Gibco, Carlsbad, CA, USA). CDDP, RIPA and 3-(4, 5-dimethylthiazol-2-yl)-2, 5-diphenyltetrazolium bromide (MTT) were purchased from Sigma-Aldrich (St Louis, MO, USA). MG132, Epoxomicin (Epox), cycloheximide (CHX), and GSK-3β inhibitor (CHIR99021) were purchased from MedChemExpress (Monmouth Junction, NJ, USA). Transfections were performed using Lipofectamine 2000 (Invitrogen, Carlsbad, CA, USA). Anti-PGC1α(M), anti-p53(M), anti-p21(M), and anti-Nrf1(M) antibodies were from Santa Cruz Biotechnology (Santa Cruz, CA, USA). Anti-Bcl-2 (R), anti-Mcl-1(R), anti-Bax (R) and anti-phospho-Akt (phospho T315/316/312) were from Abcam (Cambridge, MA, USA). Anti-cleaved caspase-3 (R), anti-GSK-3β (R), anti-phospho-GSK-3β (phospho Ser9, R), and anti-AKT (R) antibodies were from Cell Signaling Technology (Danvers, MA, USA). Anti-ubiquitin (R) and anti-actin (M) antibodies were from Proteintech (Chicago, IL, USA).

### Non-Small Lung Cancer Tissue Microarray and Immunohistochemistry

Tissue microarrays of 90 lung cancer tumors and their corresponding adjacent non-cancer tissues were obtained from Shanghai Outdo Biotech Co., Ltd. (Shanghai, China). Immunohistochemical (IHC) staining was carried out on 5-μm-thick sections of the abovementioned tissues to assess PGC1α and p53 expression. DAPI was used to stain nuclei. Images were acquired using an Aperio slide scanner and analyzed by ImageScope software (Aperio, Shanghai Outdo Biotech, China). For IHC scoring, the percentage (0, 0%; 1, 1–25%; 2, 26–50%; 3, 51–75%; and 4, >75%) of stained tumor cells was multiplied by the intensity (0, 1, 2, or 3) to achieve a score between 0 and 12.

### Cell Viability Assay

Cells (8,000 cells per well) were seeded in 96-well-plates and transfected with a PGC1α-shRNA plasmid and/or treated with CDDP for 24 h. MTT reagent was added and cells were incubated for 4 h. Formazan crystals were dissolved in 150 μL of dimethyl sulfoxide and the optical density at 570 nm was recorded by an enzyme-linked immunosorbent assay reader after the plate was shaken for 5 min.

### ATP Production

Cells were lysed with a lysis buffer, and then centrifuged (10,000 × g for 2 min) at 4°C. The level of ATP production was determined by mixing 10 μL of the supernatant with 100 μL of luciferase reagent (ATP Bioluminescence Assay Kit, Beyotime Technology, Shanghai, China). The emitted light was measured using an Omega luminometer (BMG Labtech, Ortenberg, Germany). Measurements were normalized to the protein concentration.

### Plasmids and Transfections

A full-length human p53 expression vector was constructed by subcloning a full-length *p53* cDNA fragment into pcDNA3.1 vector (Genechem, Shanghai, China). shRNA sequences targeting human *PGC1*α and a non-target sequence were constructed by Genechem. The PGC1α shRNA sequences used were: PGC1α shRNA 1: 5′-GTT-ATA-CCT-GTG-ATG-CTT-T-3′; PGC1α shRNA 2: 5′-CAG-CGA-AGA-TGA-AAG-TGA-T-3′; PGC1α shRNA 3: 5′-AGA-GTA-TGA-CGA-TGG-TAT-T-3′; and the non-target shRNA (Scramble) sequence was 5′-TTC-TCC-GAA-CGT-GTC-ACG-T-3′. Taking 6-well-plate as an example, the amount of plasmid is 4 μg/per well. Cells were transfected using Lipofectamine 2000 (Invitrogen) according to the manufacturer's instructions.

### Western Blotting

Whole-cell lysates were prepared and quantified according to standard protocols. Lysates diluted with 5 × SDS-PAGE loading buffer were boiled at 95°C for 10 min and separated by SDS-PAGE, and then electrophoretically transferred to polyvinylidene difluoride membranes. The membranes were blocked with 5% milk followed by successive incubation with primary antibodies and peroxidase-conjugated secondary antibodies. The bands were visualized using Pierce ECL Western Blot Substrate (Thermo Scientific, Waltham, MA, USA).

### RT-PCR and qRT-PCR

Total RNA was extracted using TRIzol Reagent (Invitrogen) according to the manufacturer's protocol. The reverse transcription reaction and PCR were performed using the SuperScript RT-PCR kit (Thermo Scientific). The target DNA fragments were amplified with their corresponding primers: *ACTB*: 5′-ATATCGCGTCGCTGGTCGTC-3′ (forward) and 5′-AGGATGGCGTGAGGGAGAGC-3′ (reverse); *PPARGC1A*: 5′-CAGAGAGTATGAGAAGCGAGAG-3′ (forward) and 5′-AGCATCACAGGTATAACGGTAG-3′ (reverse). The amplified products were either detected by PCR or separated by 2% agarose gel and detected using ultraviolet light. qRT-PCR was performed using the MX3000P instrument (Agilent, USA).

### Real-Time Cell Analysis (RTCA)

The cell growth status was monitored by the RTCA S16 System (ACEA Biosciences, San Diego, CA, USA), as previously reported (21).

### Detection of Protein Half-Life

For the protein half-life assay, cells were treated with 200 μM CHX (MedChemExpress) after transfection with p53 and collected at different time points. Then, cells were lysed for western blot analysis.

### Co-immunoprecipitation

Cells were lysed with NP40 lysis buffer plus protease inhibitors. Equal amounts of protein lysates were incubated with the indicated antibodies overnight at 4°C (2 μg antibody per 300–500 μg protein), followed by incubation with 30 μL of protein A/G agarose beads (Beyotime Biotechnology). The next day, the beads were rinsed three times with PBS, resuspended in 5 × SDS-PAGE loading buffer, boiled at 95°C for 10 min and centrifuged. The proteins in the supernatant were analyzed by western blot analysis.

### Flow Cytometry

Cells were seeded in 6-well-plates and treated with various reagents as indicated. Cells were then harvested and stained with Annexin V-FITC and propidium iodide (PI) (Annexin V Apoptosis Detection Kit, BD Pharmingen, San Jose, CA, USA) to measure cellular apoptosis. The mitochondrial membrane potential (MMP) was determined using the Mitochondrial Membrane Potential Assay Kit (Beyotime Biotechnology). ROS production was evaluated by DCFH-DA (Beyotime Biotechnology). Analysis was performed using a BD Accuri C6 flow cytometer (BD Bioscience) or a BD FACSAria II (BD Bioscience). Data analysis was performed using FlowJo v10 or BD Accuri C6 Software.

### Fluorescence Microscopy

Cells were seeded on glass cover slips in a 24-well-plate and treated as indicated. Then, cells were washed with PBS, fixed with 4% paraformaldehyde for 20 min and permeabilized with 0.1% Triton X-100 for 8 min. After blocking with 5% bovine serum albumin (BSA) for 30 min, cells were incubated with primary antibody overnight at 4°C. After PBS washing, the cells were incubated at room temperature for 1 h in the dark with FITC/Texas Red-conjugated secondary antibodies (Proteintech). The images were observed on an Echo-lab Revolve microscope (CA, USA).

### *In vivo* Xenograft Experiments

Animal experiments were performed following the National Institutes of Health Guide for the Care and Use of Laboratory Animals, with the approval of the Animal Welfare and Ethics Group of the Laboratory Animal Science Department, Jilin University (Changchun, China). H1299 cells (3 × 10^6^) were subcutaneously injected into the upper flank of 4-week-old female BALB/C nude mice purchased from the Beijing Vital River Laboratory Animal Technology (Beijing, China). Two weeks after the injection, the mice were randomly divided into four groups (four mice per group): control, CDDP+Scr-shRNA, PGC1α-shRNA, and CDDP+PGC1α-shRNA. CDDP (3 mg/kg) was intraperitoneally administered every 2 days and 100 μL of PGC1a-shRNA plasmid formulated with attenuated Salmonella Typhi strain Ty21a (1 × 10^7^ CFU/100 L) were injected every week. The body weight and tumor volume were recorded every 2 days. After 21 days of treatment, mice were sacrificed and tumors were dissected, weighed, and photographed.

### Tunel Assay

Mouse tumor tissues were fixed in 4% (w/v) paraformaldehyde, dehydrated in ethanol gradient, and embedded in paraffin. Samples were then cut into 3-μm sections using a Leica microtome. Terminal deoxynucleotidyl transferase dUTP nick end labeling (TUNEL) assay was carried out according to the manufacturer's instructions (Roche Ltd., Mannheim Germany). Sections were analyzed using an inverted fluorescence microscope (Olympus, Tokyo, Japan).

### Statistical Analysis

Data are expressed as the mean ± SD. ^*^P < 0.05, ^**^P < 0.01, and ^***^P < 0.001 were considered statistically significant. Statistical analysis was performed with GraphPad Prism 5 (La Jolla, CA, USA). All experiments were repeated at least three times.

## Result

### The Expression of p53 and PGC1α Correlates With the Survival Rate of Non-Small Cell Lung Cancer

**Figure 1:**
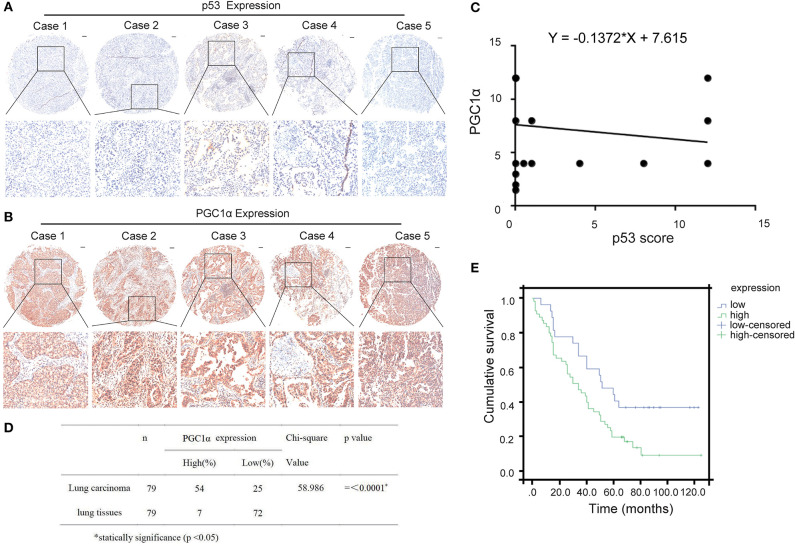
Expression of PGC1α and p53 in human non-small lung cancer tissues. **(A,B)** Immunohistochemical staining of PGC1α and p53 in NSCLC tissue microarrays (40×). Typical staining is shown in the adjacent rows (100×). **(C)** PGC1α expression negtively corrected with p53 expression in NSCLC tissues analyzed by spearman correlation (Rho = −0.341, *P* < 0.01). **(D)** Expression of PGC1α in lung carcinoma and lung tissues (*P* = 0.00). **(E)** Kaplan–Meier survival curve shows significant correlation between high PGC1α expression and low survival in human NSCLC.

Previous studies have reported that p53 binds to the promoter region of *PGC1*α and regulates its activity (22). To determine the relationship between p53 and PGC1α, we investigated the expression of p53 and PGC1α in human NSCLC tissues from 90 patients by immunohistochemical staining. Spearman correlation analysis showed that the p53 expression negatively correlated with PGC1α expression (Rho = −0.341, *P* < 0.01; Figures 1A–C). Furthermore, the tumor tissues had increased PGC1α expression compared with the normal lung tissues (Figure 1D), and the increased expression of PGC1α was associated with low survival rate of NSCLC as assessed by Kaplan–Meier analysis (*P* = 0.017; Figure 1E). Taken together, these results suggest that there is negative relationship between p53 and PGC1α, and that PGC1α may be a potential target for treatment of NSCLC with low p53 expression.

### Chemosensitivity to CDDP Is Determined by Both p53 and PGC1α Expression in NSCLC Cells

**Figure 2:**
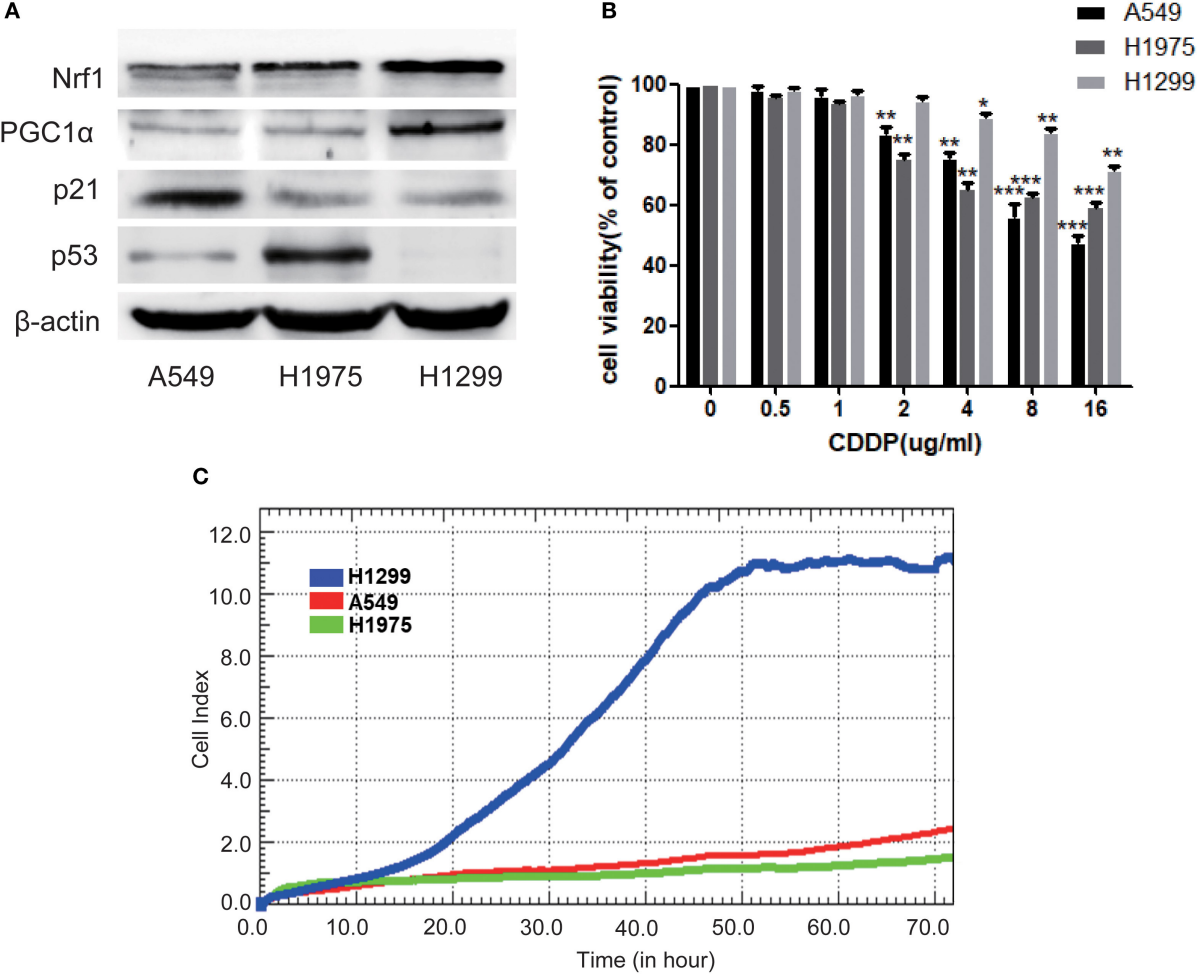
The chemosensitivity to CDDP is determined by both the expression of p53 and PGC1α in NSCLC cells. **(A)** The expression of PGC1α and its downstream target Nrf1, and of p53 and its downstream target p21 in A549 (p53 wild type), H1975 (p53 mutant), and H1299 (p53-null) cells was examined by western blotting. **(B)** A549, H1975, and H1299 cells were treated with different doses of CDDP for 24 h. Cell viability was determined by the MTT assay. Data are the mean ± SD, *n* = 3, *P < 0.05, **P < 0.01, ***P < 0.001, compared with the respective controls. **(C)** Time-dependent cell growth curve of human non-small lung cancer cells. The cell suspensions were transferred to E-Plates and placed on the RTCA reader for real-time monitoring every 5 min for the duration of the assay. The number of cells inside the well is displayed as the Cell Index.

To further investigate the relationship between p53 and PGC1α in NSCLC cells, we examined the expression of PGC1α in cells with different variants of p53, that is, A549 (p53 wild type), H1975 (p53 mutant), and H1299 (p53-null). We found that compared with A549 and H1975, H1299 had increased expression of PGC1α and its downstream target, Nuclear factor 1 (Nrf1) (Figure 2A). Mutations or deletions in the *TP53* gene primarily result in impaired tumor suppressor function (23). Notably, loss of p53 function is linked to resistance to chemotherapeutic agents (24), while increased PGC1α expression leads to drug resistance by upregulating oxidative phosphorylation (OXPHOS) (25). Next, we determined the sensitivity to CDDP using the MTT assay, which revealed that the cell viability of H1299 cells was significantly higher than that of A549 and H1975 cells after CDDP treatment (Figure 2B). Furthermore, RTCA indicated a shorter time for H1299 cells to reach the logarithmic phase, compared with the other cells (Figure 2C). These results suggest that H1299 (p53-null) cells have high PGC1α expression, which decreases their sensitivity to CDDP treatment.

### p53 Affects the Stability of PGC1α Through the Ubiquitin Proteasome Pathway

**Figure 3:**
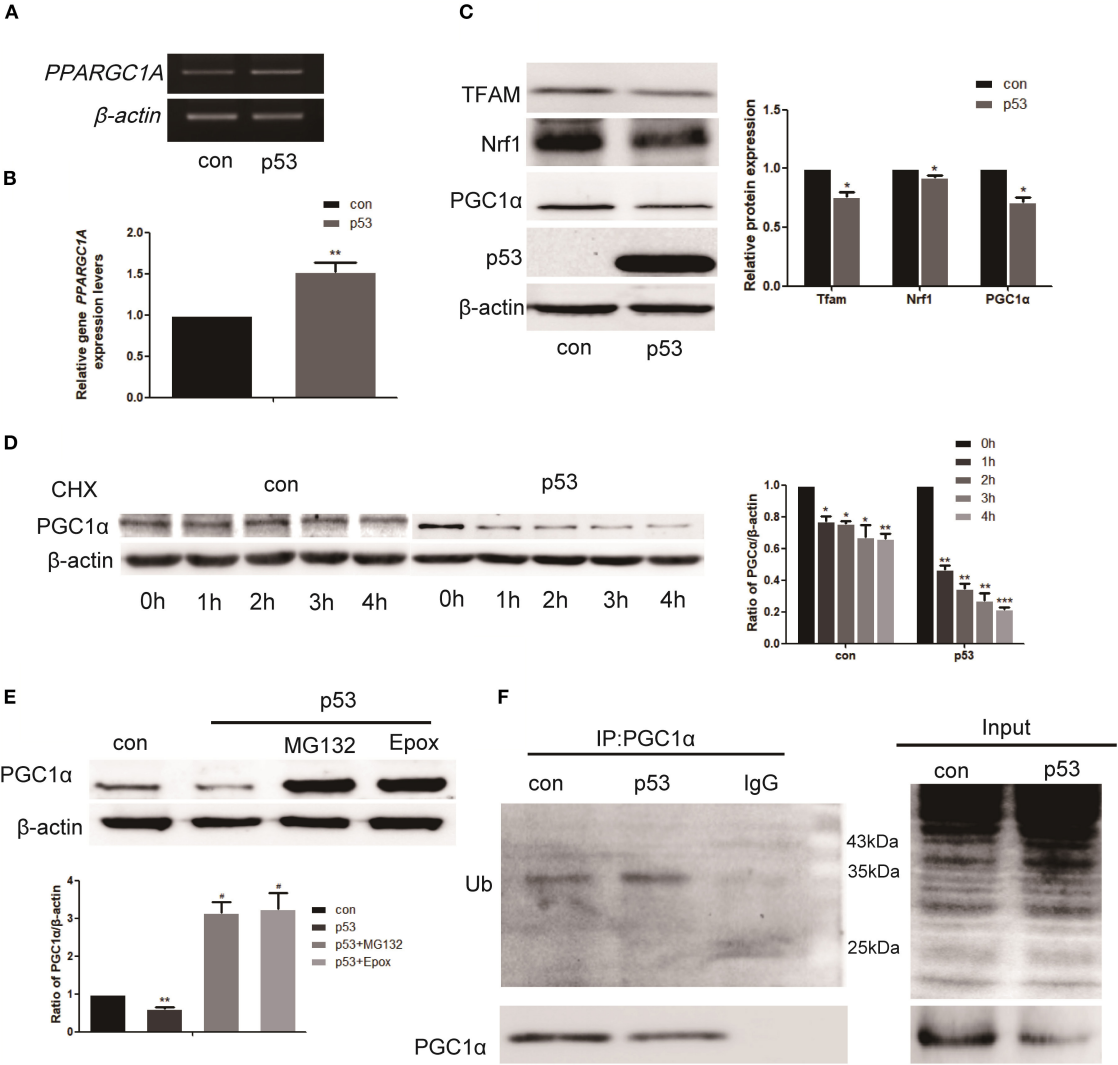
p53 affects the stability of PGC1α through the ubiquitin proteasome pathway. H1299 cells were transfected with a p53 overexpression construct or pcDNA3.1 (empty vector as the control group) for 48 h. **(A,B)** Relative *PGC1*α expression was measured by RT-PCR and qRT-PCR. Data are the mean ± SD, *n* = 3, **P < 0.01, compared with the control. **(C)** Western blot analysis of the expression of PGC1α and its downstream targets Tfam and Nrf1. Data are the mean ± SD, *n* = 3, *P < 0.05, compared with the control. **(D)** H1299 cells transfected with p53 or empty vector for 24 h were treated with 200 μM CHX, collected at the indicated time points, and analyzed by western blotting. Data are the mean ± SD, *n* = 3, *P < 0.05, **P < 0.01, ***P < 0.001, compared with the control. **(E)** H1299 cells were treated with the proteasome inhibitors, 2 μM MG132, and 50 nM Epox, for 18 h after transfection with p53. The expression of PGC1α was examined by western blotting. Data are the mean ± SD, *n* = 3, **P < 0.01, compared with the control, ^#^*P* < 0.05, compared with the p53 group. **(F)** H1299 cells were transfected with p53 or empty vector for 48 h. Immunoprecipitation was performed with anti-PGC1α antibodies followed by western blotting using anti-ubiquitin and anti-PGC1α antibodies.

**Figure 4:**
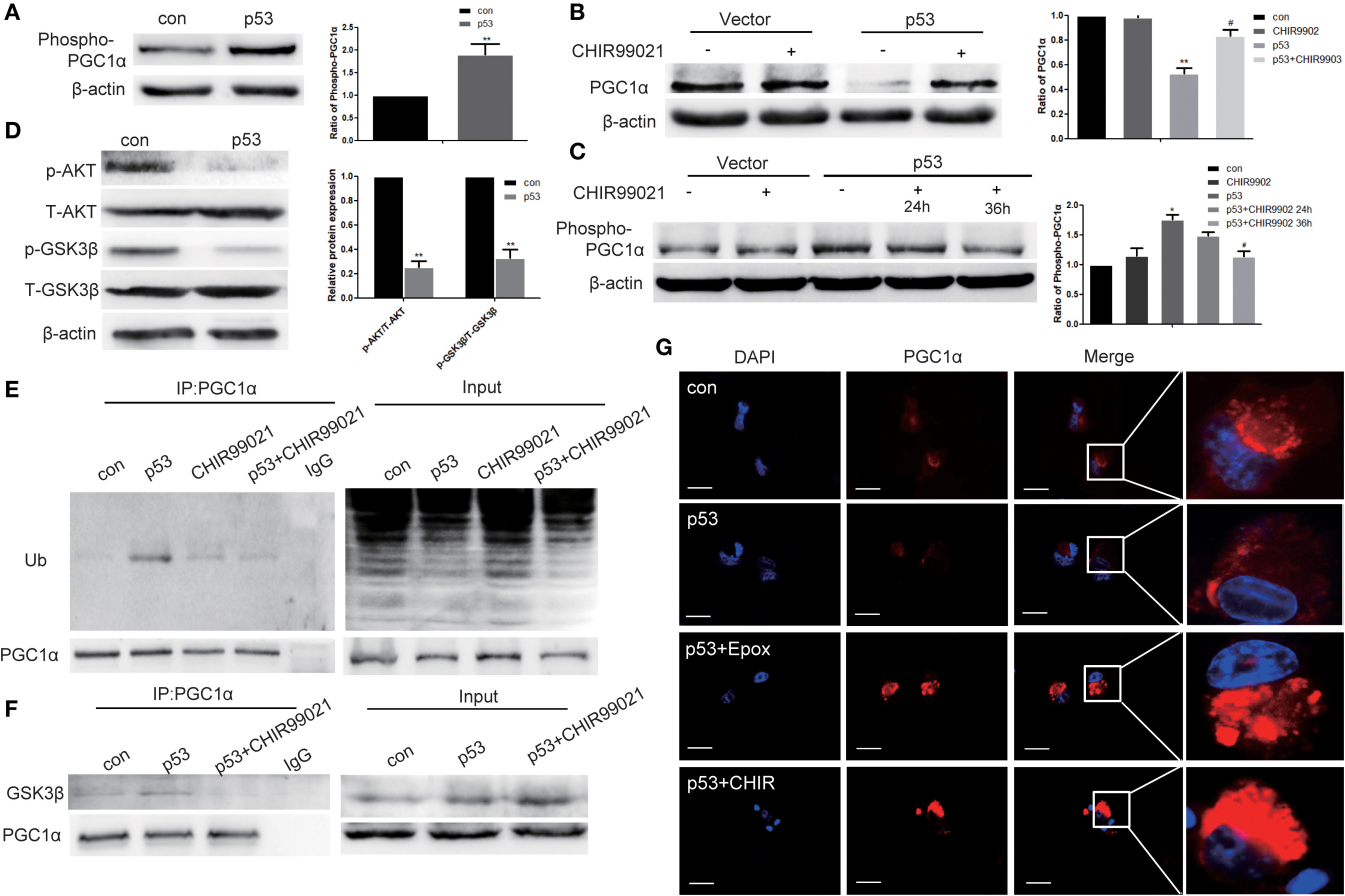
p53 promotes the degradation of PGC1α by the ubiquitin proteasome pathway via AKT/GSK-3β-dependent phosphorylation. H1299 cells were transfected with p53 or empty vector for 48 h. The expression of phospho-PGC1α **(A)** and p-AKT, T-AKT, p-GSK-3β, T-GSK-3β **(D)** were measured by western blotting. Data are the mean ± SD, *n* = 3, **P < 0.01, compared with the control. H1299 cells were treated with the GSK-3β inhibitor, 5 μM CHIR99021, for 24 h or 36 h after transfection with p53 or empty vector. **(B,C)** Western blotting determined the expression of PGC1α and phospho-PGC1α. Data are the mean ± SD, *n* = 3, *P < 0.05, **P < 0.01, compared with the control; ^#^*P* < 0.05, compared with the p53 group. **(E,F)** Immunoprecipitation was performed with the anti-PGC1α antibody followed by western blotting using anti-ubiquitin, anti-GSK-3β, and anti-PGC1α antibodies. **(G)** H1299 cells were treated with 50 nM Epox for 18 h or 5 μM CHIR99021 for 36 h after transfection with p53 or empty vector. PGC1α expression was determined by immunofluorescence staining (magnification ×400).

As shown above, there was a negative correlation between p53 and PGC1α expression. To investigate how p53 regulates PGC1α, we examined the effect of p53 on PGC1α at both the mRNA and protein levels. We first demonstrated that p53 was successfully overexpressed (Supplementary Figure 1). The RT-PCR and qRT-PCR results showed that p53 promoted the expression of *PPARGC1A* at the mRNA level (Figures 3A,B). However, there was a decrease in the expression of PGC1α protein and the downstream proteins, Nrf1 and Mitochondrial transcription factor A (Tfam), after p53 overexpression (Figure 3C). Consistently, the immunofluorescent staining of PGC1α was also decreased (Figure 4G). These results prompted us to ask whether p53 affects the stability of PGC1α. Hence, we examined the degradation rate of PGC1α by translation inhibition experiments using Cycloheximide (CHX), which is widely used for exploring protein degradation (26, 27). The level of PGC1α in p53-overexpressing H1299 cells decreased significantly at 1 h compared with the control group (Figure 3D), indicating that p53 decreased the stability of PGC1α.

As the proteasome is one of the principal mechanisms for specific depletion of proteins, we used proteasome inhibitors (MG132 and Epox). Western blot analysis showed that the PGC1α levels increased in the presence of MG132 and Epox (Figure 3E). Proteins degraded by the proteasome are polyubiquitinated on their polypeptide chains (28). Therefore, we performed immunoprecipitation of PGC1α to investigate the presence of PGC1α-ubiquitin conjugates in H1299 cells. Western blot analysis demonstrated that the ubiquitinated form was moderately enriched in precipitates from cells overexpressing p53 (Figure 3F). These data indicate that p53 decreased the stability of PGC1α by the ubiquitin proteasome pathway.

### Degradation of PGC1α by p53 Requires AKT/GSK-3β-Dependent Phosphorylation

Proteins are often phosphorylated before being recognized by the ubiquitin-proteasome pathway. Western blotting showed increased phospho-PGC1α after transfection with p53 (Figure 4A). Previous studies have confirmed that GSK-3β-mediated phosphorylation primes BMAL1 for subsequent degradation via proteasomal degradation. We asked whether p53 induced PGC1α degradation through GSK-3β. In the presence of GSK-3β inhibitor (CHIR99021), the decreased levels of PGC1α after transfection with p53 was reversed (Figure 4B). Furthermore, the increased expression of phospho-PGC1α decreased after CHIR99021 treatment (Figure 4C). GSK-3β activity is regulated by inhibitory phosphorylation and p53 promotes GSK-3β activity by inhibiting AKT. Next, we measured the phosphorylation levels of GSK-3β and AKT after transfection with p53. Western blot analysis revealed that p53 decreased the phosphorylation of both AKT and GSK-3β, indicating that GSK-3β was activated (Figure 4D).

To further confirm whether GSK-3β was directly involved in PGC1α-ubiquitin degradation, we conducted immunoprecipitation experiments using cells transfected with p53 in the absence or presence of GSK-3β inhibitor. The results showed that the enhanced PGC1α ubiquitination after transfection with p53 was reversed by GSK-3β inhibitor (Figure 4E). Moreover, p53 slightly increased the co-immunoprecipitation of GSK-3β with PGC1α, and this association was impaired in the presence of GSK-3β inhibitor (Figure 4F). To further confirm this, we performed immunofluorescence experiments, which showed that PGC1α staining was elevated after treatment with Epox or GSK-3β inhibitor combined with p53 overexpression (Figure 4G).

### PGC1α Knockdown Combined With CDDP Promotes Apoptosis by Reducing Mitochondrial Function

**Figure 5:**
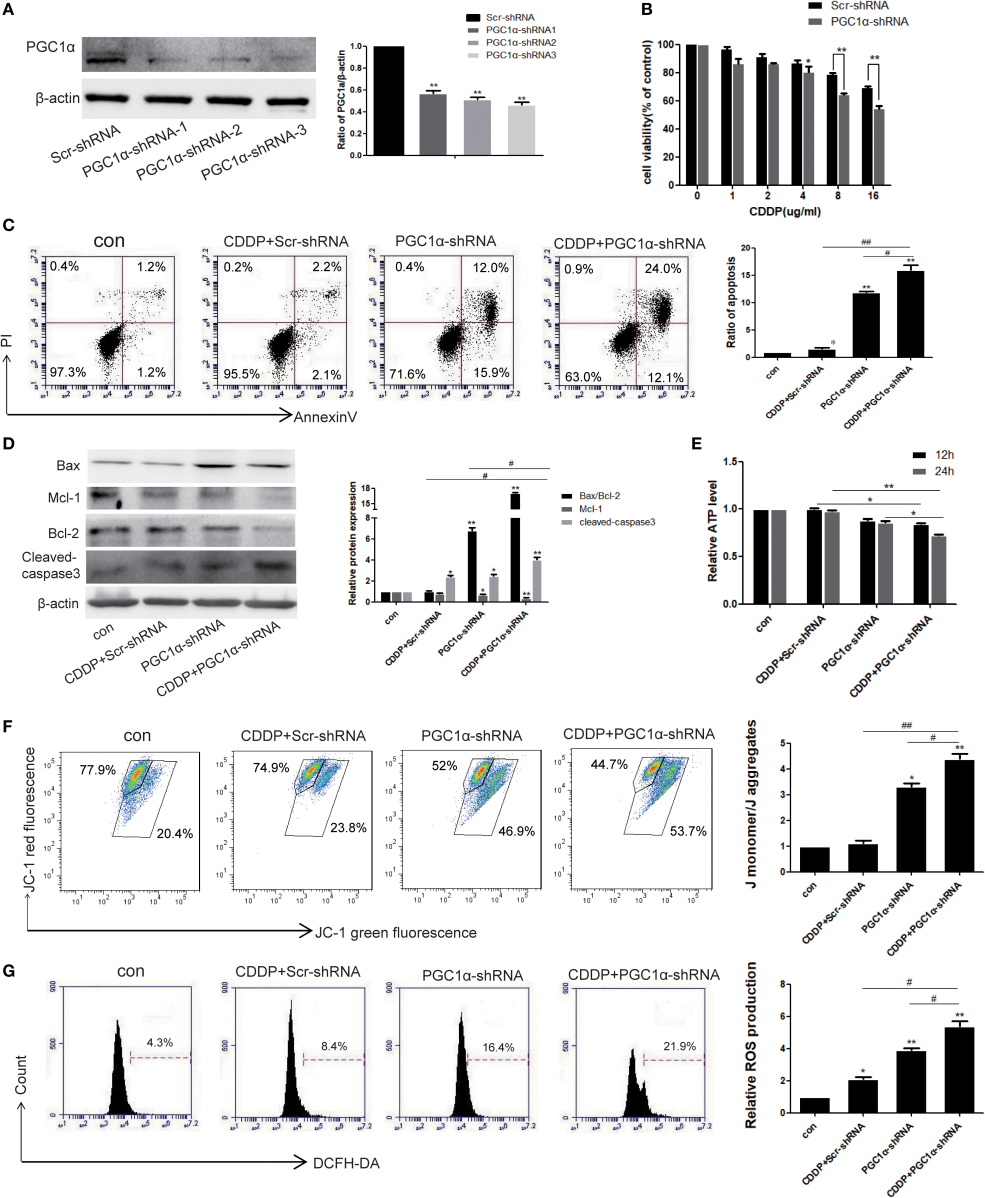
Knockdown of PGC1α combined with CDDP treatment promotes apoptosis by reducing mitochondrial function. **(A)** H1299 cells were transfected with three PGC1α-shRNA plasmids and Scr-shRNA for 48 h. Western blotting was used to analyze the knockdown efficiency. **(B)** Cell viability of transfected H1299 cells exposed to different doses of CDDP for 24 h was determined by the MTT assay. Data are the mean ± SD, *n* = 3, *P < 0.05, compared with the control, **P < 0.01, compared with the respective CDDP-treated Scr-shRNA group. **(C,D)** H1299 cells were treated with CDDP (8 μg/mL) for 24 h after transfection with PGC1α-shRNA plasmid or Scr-shRNA plasmid. Annexin V/PI staining **(C)** and western blotting analysis of Bax, Bcl2, Mcl-1, and cleaved caspase-3 **(D)** were used to detecte apoptosis. Data are the mean ± SD, *n* = 3, *P < 0.05, **P < 0.01, compared with the control; ^#^*P* < 0.05, ^##^*P* < 0.01. **(E)** ATP production in the transfected cells was determined by a kit after treatment with CDDP for 12 or 24 h. Data are the mean ± SD, *n* = 3, *P < 0.05, **P < 0.01. **(F,G)** Cells transfected with PGC1α-shRNA plasmid or Scr-shRNA plasmid were treated with CDDP (8 μg/mL) for 24 h. Cells were stained with JC-1 **(F)** or DCFH-DA **(G)**, followed by flow cytometry to evaluate the MMP and ROS level. Data are the mean ± SD, *n* = 3, *P < 0.05, **P < 0.01, compared with the control; ^#^*P* < 0.05, ^##^*P* < 0.01.

To further verify that high expression of PGC1α is associated with CDDP resistance, PGC1α was knocked down in H1299 cells by transient transfection with shRNA (Figure 5A). The MTT assay demonstrated that PGC1α knockdown increased the sensitivity of H1299 cells to CDDP compared with the Scr-shRNA group (Figure 5B). Next, after treatment with CDDP and/or transfection with PGC1α-shRNA for 24 h, we examined apoptosis by Annexin V/PI staining and western blotting. The results showed an increase in apoptosis after PGC1α knockdown or CDDP treatment, and the level of apoptosis was further increased in the combined group (Figure 5C). Additionally, the expression of the apoptotic proteins, cleaved caspase-3 and Bax, was increased and that of the antiapoptotic proteins, MCl-1 and Bcl-2, was decreased after transfection with PGC1α-shRNA or CDDP treatment, and this effect was further enhanced in the combined group (Figure 5D). Furthermore, we measured the ATP level. The results showed that transfection with PGC1α-shRNA or CDDP treatment reduced the ATP content in H1299 cells, and the combined group showed a further decrease (Figure 5E). Next, JC-1 fluorescent staining was used to measure MMP. The results showed that transfection with PGC1α-shRNA or CDDP treatment reduced the MMP, which was further decreased in the combined group (Figure 5F). We also observed more ROS production in the PGC1α-shRNA and CDDP combined group (Figure 5G). These results suggest that PGC1α knockdown combined with CDDP treatment promoted apoptosis by impairing mitochondrial function.

### Effects of PGC1α Knockdown Combined With CDDP Treatment on *in vivo* Tumor Xenografts

**Figure 6:**
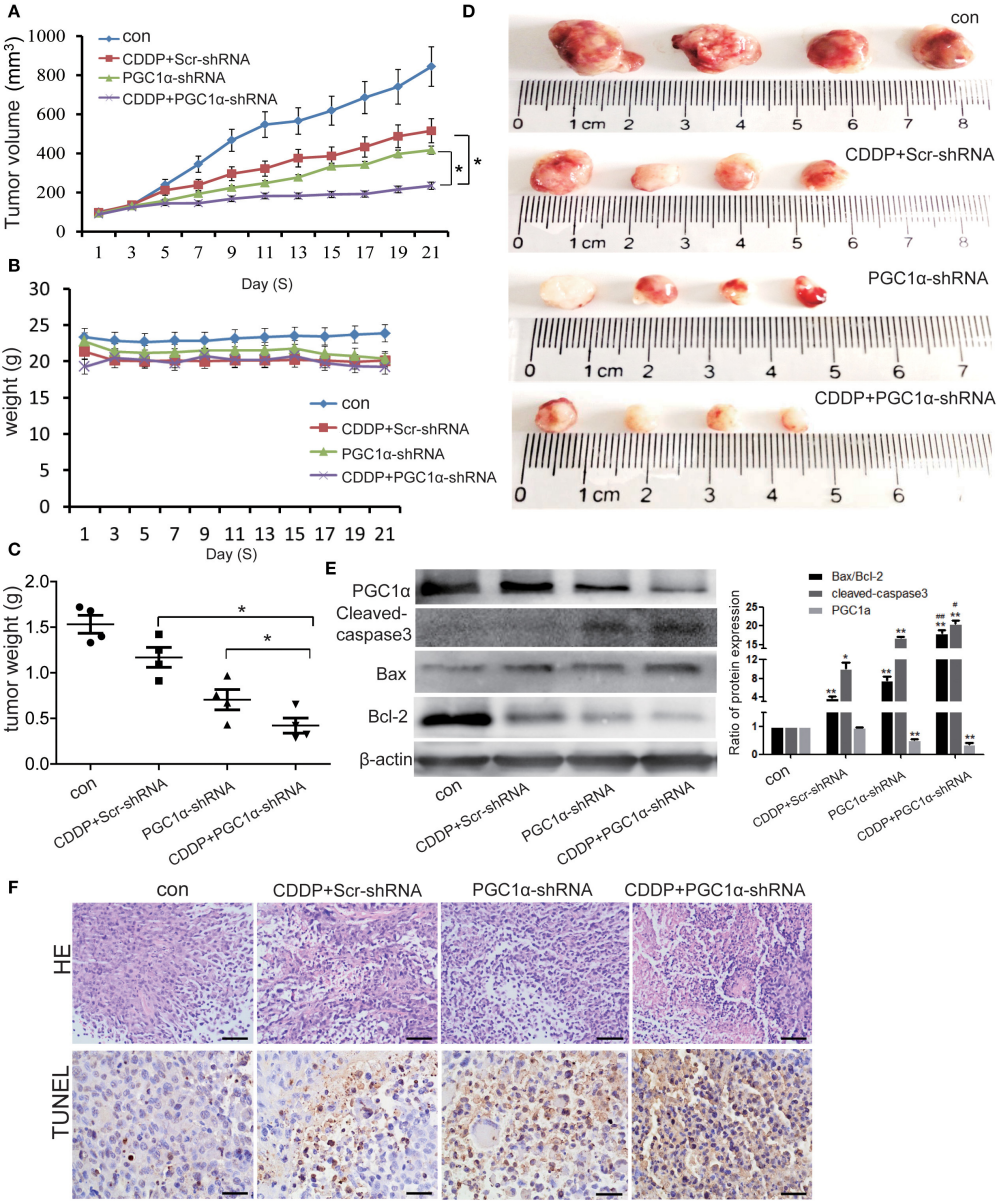
Effects of PGC1α knockdown combined with CDDP treatment *in vivo* tumor xenografts. H1299 cells were subcutaneously implanted into nude mice. Mice were treated with 3 mg/kg CDDP and intravenously injected with attenuated Salmonella Typhi strain Ty21a harboring the PGC1α-shRNA or Scr-shRNA plasmid for 21 days (*n* = 4 per group). **(A–C)** Tumor volume and body weight were measured every 2 days. Tumor volume was determined by measuring the length and width with calipers. The wet weight of the tumors was determined at autopsy. **(D)** Images of excised tumors from each treatment group. Data are the mean ± SD, *n* = 3, *P < 0.05, compared with the CDDP group. **(E)** Tumor tissues from the mouse xenograft model were lysed with RIPA buffer and the expression of PGC1α, Bcl2, Bax, and cleaved caspase-3 was analyzed by western blotting. Data are the mean ± SD, *n* = 3, *P < 0.05, **P < 0.01, compared with the control; ^#^*P* < 0.05, ^##^*P* < 0.01, compared with the CDDP-shRNA or PGC1a-shRNA groups. **(F)** Representative images of the TUNEL assay performed on mouse xenograft tumor specimens. Scale bar, 50 μm.

To examine the effects of PGC1α knockdown and CDDP treatment *in vivo*, we established tumor xenografts by inoculating H1299 NSCLC cells in immunodeficient BALB/C nude mice. We found that PGC1α knockdown improved the effects of CDDP treatment and inhibited tumor growth (Figures 6A–C). Western blot analysis showed that the expression of the apoptotic proteins, Bax and cleaved caspase-3, was increased, while the expression of proapoptotic Bcl-2 was decreased after transfection with PGC1α-shRNA combined with CDDP treatment (Figure 6E). Moreover, TUNEL staining revealed that PGC1α knockdown combined with CDDP treatment significantly increased apoptosis compared with either treatment alone (Figure 6F), which was consistent with the *in vitro* experiments. These results further confirmed that knockdown of PGC1α combined with CDDP treatment enhanced the inhibition of NSCLC cells.

## Discussion

Mitochondria are the primary energy source for cellular function. Mitochondrial biosynthesis is a major cellular process that maintains mitochondrial functions (29). Numerous studies have identified the important roles of enhanced mitochondrial biosynthesis and energy metabolism in tumorigenesis and drug resistance (15). PGC1α, a major regulator of mitochondrial biogenesis, seems to perfectly reflect cellular energy requirements and the control of mitochondrial protein production, as increased demand for energy induces its expression (30). Previous studies have verified that p53 maintains mitochondrial biosynthesis by regulating mitochondrial DNA (31), therefore, p53 and PGC1α may play a common role in regulating mitochondrial biogenesis. Both Sahin et al. and Sen et al. have found that p53 negatively regulates PGC1α levels (22, 32), which was also verified in our study. We found a negative correlation between PGC1α and p53 expression in NSCLC tissues. Moreover, patients with high PGC1α expression have a short survival period. Further verification was performed by using NSCLC cells with different p53 backgrounds. The results showed that p53-deficient H1299 cells had higher expression of PGC1α and were less sensitive to CDDP. When p53 was overexpressed, the protein expression level of PGC1α and its downstream targets Tfam and Nrf1 was significantly decreased despite their increased gene expression. CHX experiments also confirmed that p53 promotes the protein degradation of PGC1α. Additionally, in our study, the proteasome inhibitors, MG132 and Epox, prevented most of the p53-mediated decrease in PGC1α protein levels. Therefore, we concluded that p53 negatively regulates PGC1α protein expression in NSCLC mostly by promoting its degradation.

Proteins are usually phosphorylated before being recognized by ubiquitin, which is easily recognized by the ubiquitin proteasome system (33). In our experiments, PGC1α phosphorylation and ubiquitination were significantly increased after p53 overexpression. Besing et al. have reported that phosphorylation by GSK-3β primes BMAL1 for ubiquitination and subsequent degradation (34). Hong et al. have found a new mechanism for the DNA damage-induced depletion of SOX9 that involves SOX9 phosphorylation by GSK-3β, thus targeting SOX9 for ubiquitination and proteasomal degradation (35). Notably, AKT inhibits GSK-3β activity by phosphorylating Ser9, while p53 inhibits AKT via PHLDA3 (18, 36, 37). In our experiments, p53 inhibited GSK-3β activity by inhibiting AKT. When combined with a GSK-3β inhibitor, the p53-induced increase in PGC1α phosphorylation and ubiquitination was reversed, and the decrease in PGC1α expression was reversed. Taken together, we concluded that p53 promotes the degradation of PGC1α through the AKT/GSK-3β pathway.

We demonstrated that high levels of PGC1α are associated with poor prognosis for NSCLC patients and with poor CDDP sensitivity of H1299 (p53-null) lung cancer cells. These findings are similar to the research of Vellinga et al. (14), who found that upregulating the PGC1α signaling pathway reduced the sensitivity to CDDP by transforming tumor metabolism from glycolysis to OXPHOS in colon cancer. When we expressed shRNA directed against *PGC1*α in H1299 cells, the cell sensitivity to CDDP increased, suggesting that chemoresistance of lung cancer cells with low expression of p53 is associated with high levels of PGC1α. PGC1α regulates energy metabolism and mitochondrial biogenesis primarily by coordinating with other transcription factors such as NRF1, NRF 2, and TFAM (38). In our experiments, PGC1α knockdown reduced the ATP content and the MMP in H1299 cells. This is consistent with the study of Alonso-Molero et al. on colorectal cancer, which demonstrated that decreased levels of PGC1α reduced the MMP, thus reducing chemotherapy resistance (39). A study by Do et al. using breast cancer MCF-7 cells found that decreased PGC1α expression rendered cells susceptible to oxidative stress damage by suppressing NRF2 (40). This was also demonstrated in H1299 cells, as knockdown of PGC1α increased ROS production and significantly induced apoptosis. When PGC1α knockdown was combined with CDDP treatment, these effects were stronger. When we knocked down PGC1α *in vivo* by established tumor xenografts in mice the results were consistent with the *in vitro* findings. The above experiments indicate that inhibition of PGC1α increases CDDP sensitivity and apoptosis sensitivity by reducing mitochondrial biogenesis and energy metabolism in lung cancer cells with dysfunctional p53.

In conclusion, we found that NSCLC patients with low p53 expression and high PGC1α expression had low survival rates. p53 regulates the chemotherapeutic sensitivity of tumors by regulating the stability of PGC1α via AKT/GSK-3β-mediated phosphorylation. This may be a promising therapeutic avenue for overcoming drug resistance of NSCLC patients with different p53 backgrounds.

## Data Availability Statement

The datasets in the current study are available from the corresponding author on reasonable request.

## Ethics Statement

The studies involving human participants were reviewed and approved by Ethics Clerk of Shanghai Outdo Biological Technology Co., Ltd.

## Author Contributions

XD performed cell research. YC and SG performed data curation. BY performed animal experiments. YLi and YLiu designed the research and supervised this study. XD wrote the manuscript. LS and JS reviewed and edited the draft. All authors have read and approved the final manuscript.

## Conflict of Interest

The authors declare that the research was conducted in the absence of any commercial or financial relationships that could be construed as a potential conflict of interest.

## Abbreviations

NSCLC: non-small cell lung cancer
PGC1α: peroxisome proliferator-activated receptor coactivator
CDDP: Cisplatin
MTT: 3-(4, 5-dimethylthiazol-2-yl)-2,5-diphenyltetrazolium bromide
AKT: protein kinase B
GSK-3β: glycogen synthase kinase
RTCA: real-time cell analysis.
