# Supplementary figures and images for: p53 Affects PGC1α Stability Through AKT/GSK-3β to Enhance Cisplatin Sensitivity in Non-Small Cell Lung Cancer
Source: Front Oncol. 2020 Aug 21;10:1252. doi: 10.3389/fonc.2020.01252 (PMC7471661; doi:10.3389/fonc.2020.01252)

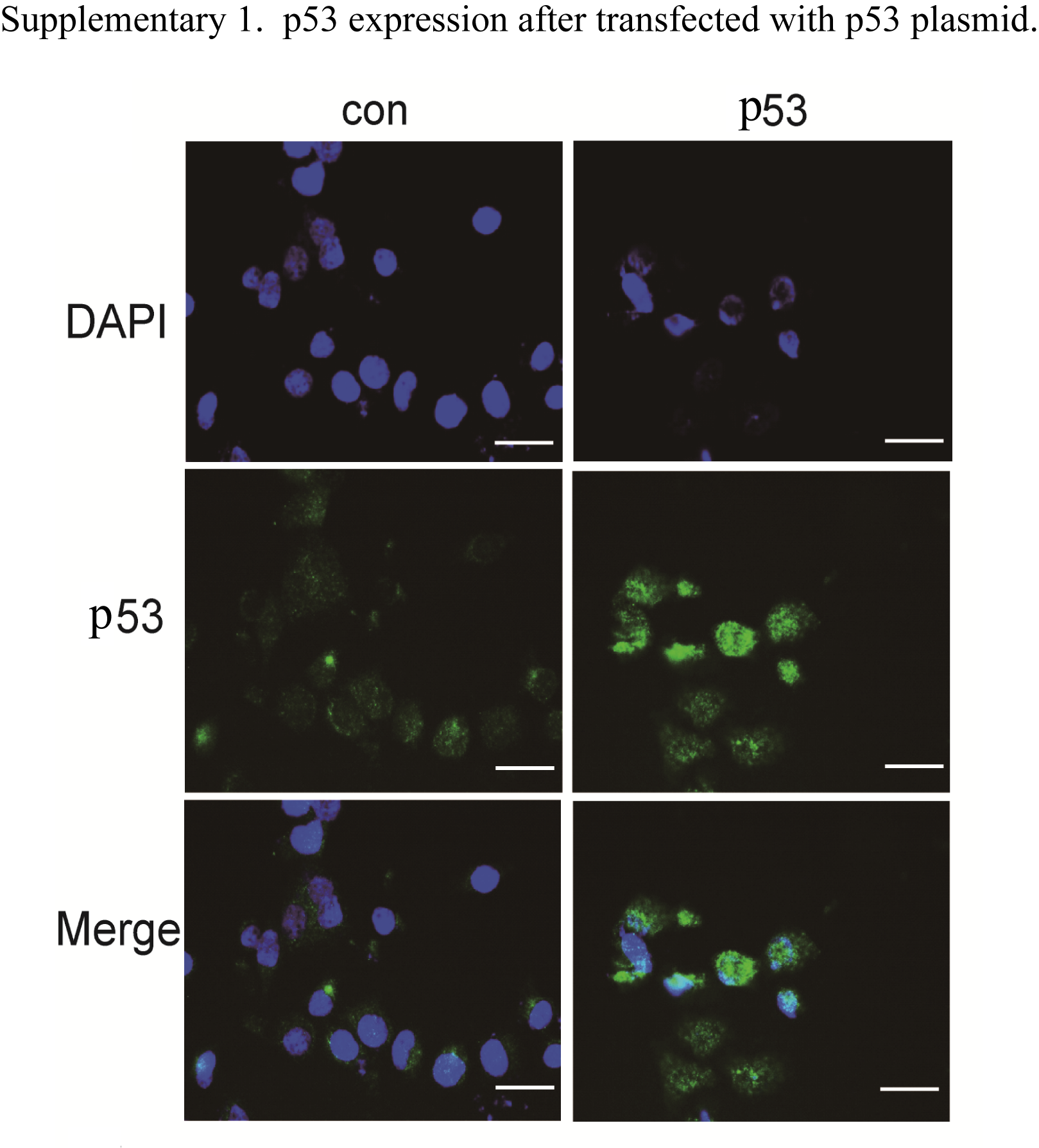

Supplement: Supplementary file 1 [file Image_1.tif]
